# A Case Report on the Dramatic Response of ^177^Lu-PSMA Therapy for Metastatic Prostate Cancer

**DOI:** 10.2174/0115734056362468250709045212

**Published:** 2025-07-18

**Authors:** Aysenur Sinem Erdogan, Haluk Sayan, Bedri Seven, Berna Okudan

**Affiliations:** 1 Department of Nuclear Medicine, Ankara City Hospital, University of Health Sciences, Ankara, Turkey; 2 Department of Radiation Oncology, Ankara City Hospital, University of Health Sciences, Ankara, Turkey; 3 Department of Nuclear Medicine, Sabuncuoğlu Şerefeddin Training and Research Hospital, University of Amasya, Amasya, Turkey

**Keywords:** Prostate-specific membrane antigen, Metastatic castration-resistant prostate cancer, Prostate cancer, ^177^Lu-PSMA, Radioligand therapy, Theranostics

## Abstract

**Introduction:**

In nuclear medicine, Prostate-specific Membrane Antigen (PSMA) is a potential target for theranostics. Offering superior diagnostic accuracy to conventional imaging in prostate cancer (PCa), Gallium-68 labeled PSMA (^68^Ga-PSMA) positron emission tomography/computed tomography (PET/CT) is considered the new standard of care in PCa management. Tumor cells identified as PSMA-avid on PET/CT imaging can be targeted and eliminated with PSMA-labeled Lutetium-177 (^177^Lu-PSMA) therapy.

**Case Presentation:**

A sixty-eight years old patient who had metastatic castration-resistant PCa was reported in this study. Prior to receiving ^177^Lu-PSMA therapy, the patient’s PSA level was 358 ng/ml, and experienced extensive bone discomfort. Following ten cycles of ^177^Lu-PSMA therapy, exceptional results were observed.

**Conclusion:**

^177^Lu-PSMA therapy is likely to result in significantly better outcomes if first- or second-line treatments preserve the patient's bone marrow reserve or if the therapy is administered at earlier stages of the disease.

## 1 INTRODUCTION

The second most prevalent type of cancer to be detected in males is Prostate Cancer (PCa) [1]. In most cases, PCa spreads to the bone, which is the main site of morbidity. However, the lymphatics, liver, and lungs are all frequently affected areas. Usually, PCa causes death due to resistance to castration levels of testosterone, which arises with the initiation of hormonal therapies. Castration-resistant PCa (CRPCa) is the term used to describe this clinical presentation [2]. The metastatic rate among patients is 19%. The typical life expectancy for these patients is approximately 14 months [3]. Although chemotherapeutic agents have been demonstrated to be more effective over time in improving survival and managing pain in metastatic castration-resistant PCa (mCRPCa), some patients still exhibit progression [4, 5]. The current standard of care for PCa management is Gallium-68 labeled prostate-specific membrane antigen (^68^Ga-PSMA) positron emission tomography/computed tomography (PET/CT), which offers superior diagnostic accuracy compared to conventional imaging. In PET/CT imaging, tumor cells that show up as PSMA-avid can be treated by injecting PSMA-labeled Lutetium-177 (^177^Lu-PSMA) radiopharmaceuticals, which eliminate PSMA-avid metastases [6, 7]. A patient who responded dramatically to ^177^Lu-PSMA therapy is presented here.

## 2 CASE REPORT

We reported a 68-year-old male patient treated with ^177^Lu-PSMA for mCRPCa. The patient had a prostate biopsy with 12-quadrant guidance. According to histopathology, the patient exhibited an extracapsular involved PCa with a Gleason Score of 9. The total serum PSA level was 4256 ng/ml at the beginning. In February 2020, ^68^Ga-PSMA PET/CT imaging was carried out for the first time. It showed widespread sclerotic skeletal and lymphatic metastases, and an aggressive PCa affecting the entire prostate gland. Due to the advanced nature of the disease, a prostatectomy was not recommended. The patient experienced extensive bone discomfort, particularly in the left upper limb. Leuprorelin/bicalutamide and docetaxel were initiated. Following the discontinuation of the docetaxel medication after 3 months due to neutropenia and leukopenia, abiraterone was prescribed. In the meantime, because of the left arm’s limited range of motion, the left shoulder was treated with palliative radiotherapy. Up until February 2021, androgen blockade and deprivation therapy were administered. With a PSA level of 358 ng/ml in February 2021, ^68^Ga-PSMA PET/CT revealed PSMA-avid PCa, abdominopelvic lymphatic, and sclerotic skeletal metastases.

**Fig. (1):**
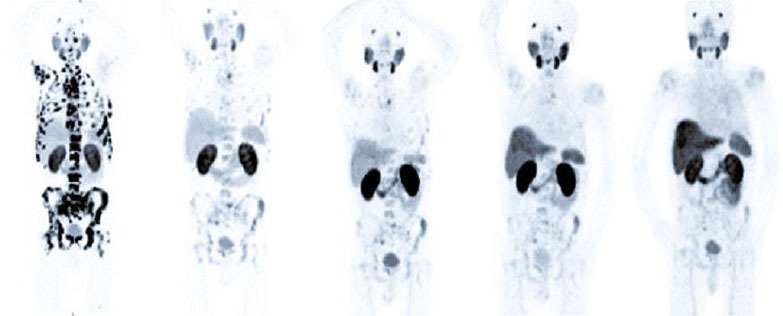
^68^Ga-PSMA PET/CT imaging was carried out from February 2021 to June 2022. Before therapies and after each of the two to three cycles, the images were obtained. The MIP-image from the baseline imaging shows PSMA-avid lymph nodes and a widespread skeletal disease. The images belong to the dates February 2021, May 2021, August 2021, October 2021, and June 2022 in order from left to right.

**Fig. (2):**
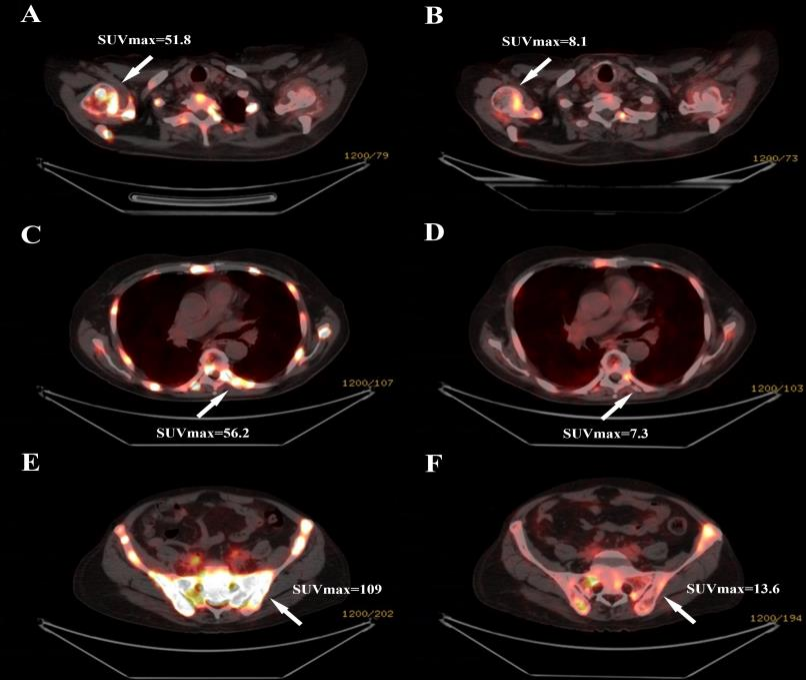
Axial fused images of ^68^Ga-PSMA PET/CT obtained before (**A**, **C** and **E**) the first and after (**B**, **D** and **F**) the sixth cycle of ^177^Lu-PSMA therapy revealed a dramatic response with significantly decreased SUVmax values of metastatic bone lesions (arrows).

In addition to abiraterone and eligard, the patient was sent to our clinic in order to receive ^177^Lu-PSMA therapy. Due to the pain, the patient’s ability to move around and his quality of life were both negatively impacted. The patient utilized transdermal fentanyl-containing tapes to reduce discomfort during the day and opioid-derived pain medications to help him sleep comfortably at night in order to manage his pain. The patient weighed 65 kg, and each treatment involved the administration of 7.4 GBq (200 mCi) of ^177^Lu-PSMA radiopharmaceutical per cycle at 6-week intervals [8]. Consequently, following the first treatment, the patient’s bone pain significantly decreased. After 4 cycles of ^177^Lu-PSMA therapy, the patient discontinued utilizing transdermal analgesic patches and opioid-derived pain medications. Between cycles 4 and 8, the patient continued to take non-steroidal anti-inflammatory drugs for mild pain. After cycle 8, the patient stopped taking the prescription for pain relief. In mid-May 2021, a ^68^Ga-PSMA PET/CT imaging was carried out after the 2nd treatment. The total PSA level dropped to 72.62 ng/ml. Following 10 therapy cycles, the total serum PSA level decreased to 2.71 ng/ml from 328 ng/ml. A significant decline in PSA from baseline was consistent with a corresponding regression in PSMA expression in metastases over time. The patient’s quality of life improved as his discomfort decreased. ^68^Ga-PSMA PET/CT imaging in March 2022, 5 weeks following the tenth cycle, revealed that the prostate gland’s aggressive cancer had completely disappeared. Likewise, PSMA-avid regional and non-regional lymph nodes, as well as extensive, widely distributed skeletal disease, responded in every disease location (Figs. **1** and **2**). The maximal standardized uptake value (SUVmax) in ^68^Ga-PSMA PET/CT dropped from 108.9 to 4.1 during the course of the ^177^Lu-PSMA therapy, which is in line with a remarkable biochemical response in PSA levels.

The therapy was well-tolerated, and laboratory tests performed at regular intervals revealed no hematological or renal toxicities.

## 3 DISCUSSION

We report a patient with mCRPCa who has responded dramatically to ^177^Lu-PSMA therapy, achieving pain control that prolongs his life and improves his quality of life. The use of ^177^Lu-therapy for mCRPCa patients has increased recently [9]. A successful therapeutic outcome largely depends on selecting the right patient [10]. According to the guidelines, commencing treatment requires adequate prior therapy, PSMA expression on PET/CT, as well as normal renal function and bone marrow reserve [8, 11].

In this case, following 10 cycles of ^177^Lu-PSMA therapy, serum total PSA level decreased by 99% from baseline.

**Fig. (3):**
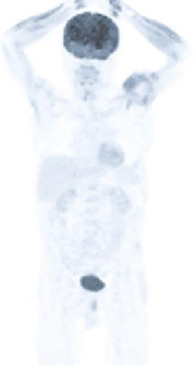
^18^F-FDG PET/CT imaging before ^177^Lu-PSMA therapy. The absence of an FDG-avid lesion was shown.

Ahmadzadehfar*et al.* demonstrated that even though the first cycle did not show any noticeable PSA response, a 50% PSA response could occur in subsequent cycles [12]. According to Wrenger *et al.*, blood values associated with poor survival had lower levels of hemoglobin prior to therapy and higher levels of LDH and ALP [13]. Additionally, liver metastases are associated with a lower chance of survival, indicating a poor prognosis. Our patient did not have any solid organ metastases, such as those to the liver or lungs [14]. Before ^177^Lu-PSMA therapy was initiated, dual imaging was performed using fluorine-18-labeled fluoro-2-deoxy-d-glucose (^18^F-FDG) PET/CT and ^68^Ga-PSMA PET/CT [15]. A lesion with accumulation of FDG but no PSMA accumulation was not observed in these imaging examinations. The lack of FDG uptake may be a contributing cause to the powerful response of the disease to therapy with ^177^Lu-PSMA (Fig. **3**). In order to contribute to the literature, we informed the patient that we would share this therapy response, and we obtained his permission. The patient died unexpectedly in 2022. Unfortunately, his death was caused by cardiovascular failure induced by COVID-19.

## CONCLUSION

The patient was referred to our clinic to receive therapy with 177Lu-PSMA by a multidisciplinary committee, despite having normal kidney function and an intact blood marrow reserve. We successfully achieved an outstanding response by using ^177^Lu-PSMA therapy in a way that would minimize toxicity in the following cycles. The effectiveness of ^177^Lu-PSMA in treating metastatic castration-resistant prostate cancer and the usefulness of ^68^Ga-PSMA PET/CT imaging for patient screening and efficacy assessment were both illustrated in this case. However, further research with a larger sample is needed to evaluate treatment and long-term follow-up in patients.

## Data Availability

All data generated or analyzed during this study are included in this published article.

## LIST OF ABBREVIATIONS

PSMA: Prostate-specific membrane antigen
PCa: Prostate cancer
^68^Ga-PSMA: Gallium-68 labeled PSMA
PET/CT: Positron emission tomography/computed tomography
^177^Lu-PSMA: PSMA-labeled Lutetium-177
CRPCa: Castration-resistant PCa
mCRPCa: Metastatic castration-resistant PCa
SUVmax: Maximal standardized uptake value
^18^F-FDG: Fluorine-18 labeled fluoro-2-deoxy-d-glucose

## STANDARDS OF REPORTING

CARE guidelines were followed.
